# Prognostic Value and Immune Infiltrates of ABCA8 and FABP4 in Stomach Adenocarcinoma

**DOI:** 10.1155/2020/4145164

**Published:** 2020-06-27

**Authors:** Ya Guo, Zhong Wei Wang, Wang Hui Su, Jing Chen, Ya Li Wang

**Affiliations:** Department of Radiation Oncology, The Second Affiliated Hospital, Xi'an Jiao tong University, Xi'an, 710004 Shaanxi, China

## Abstract

**Background:**

Stomach adenocarcinoma (STAD) is a common malignancy worldwide with poor prognosis. Therefore, it is important to identify a valuable prognostic biomarker for STAD. The aim of present study was to identify novel prognostic biomarkers for STAD and evaluate the potential role of hub genes in STAD.

**Methods:**

Gene Expression Profiling Interactive Analysis (GEPIA) and Cancer RNA-Seq Nexus were performed to identify differentially expressed genes (DEGs). Subsequently, hub genes were selected by a Venn diagram, and the expression of key genes was confirmed by UALCAN database. Furthermore, survival analysis of these hub genes was performed using Oncolnc and Human Protein Atlas (HPA) database. Gene alteration status of hub genes was assessed by cBioPortal. Finally, we investigated the association between hub genes and immune cell infiltration in STAD through the Tumor Immune Estimation Resource (TIMER) and GEPIA database.

**Results:**

Three common hub genes were obtained, including 2 downregulated DEGs (ABCA8 and FABP4) and one upregulated DEG (SLC52A3). Furthermore, increased expression of ABCA8 and FABP4 and decreased expression of SLC52A3 were correlated with poor prognosis. Meanwhile, three hub genes showed genetic alterations in various datasets of STAD. Finally, our results showed that ABCA8 and FABP4 displayed a positive correlation with immune infiltration, especially in M2 macrophages.

**Conclusions:**

The results of this study suggest that ABCA8 and FABP4 may be used as prognostic biomarkers and correlated with immune infiltration in STAD.

## 1. Introduction

Stomach adenocarcinoma (STAD) is the fifth common malignancy and the third major cause of cancer-related mortality worldwide [1]. Most patients with STAD are diagnosed at the advanced stage of the disease. The 5-year survival rate of these patients is very low, and the prognosis of STAD remains poor [2, 3]. Therefore, it is urgent to identify a new valuable prognostic marker for STAD.

Increasing evidence indicates that the immune cell infiltration plays a key role in the prognosis of tumors [4, 5]. A previous study shows that T cell infiltration is associated with the prognosis of colorectal cancer [6]. In gastric cancer or colorectal cancer, NK cell infiltration is associated with a favorable prognosis [7]. Macrophages are the most abundant immune cell in TME. Macrophages are classified into M1 and M2 categories, which play different roles in regulating the development and progression of Gastric Carcinoma [8, 9]. M1 macrophages release proinflammatory molecules such as TNF-*α*, IL-6, IL-12, and IL-8, resulting in primarily anticancer responses. However, M2 macrophages secrete Th2 cytokines, including IL-4, IL-13, and IL-10 to stimulate Th2 immune responses and activate regulatory T (Treg) cells [1, 10]. M2 macrophages promote tumor metastasis, contributing to the poor prognosis of gastric cancer [11]. Recent studies indicated that the deletion of the gene in tumor-infiltrating macrophages plays anticancer roles through inhibition of an immune suppression mechanism and is correlated with favorable prognosis [12]. In STAD, NRP1 expression may serve as an effective prognostic biomarker by predicting the infiltration of M2 macrophages [1]. It has been reported that SUPV3L1 and SLC22A17 affect immune cell infiltration, leading to the different prognosis of patients in gastric cancer [13]. In stomach adenocarcinoma, VGLL3 is identified as a novel prognostic biomarker and correlated with immune infiltrates [14]. However, the association between gene expression, the infiltration of immune cells, and prognosis has not been completely understood. Therefore, in the present study, we performed a comprehensive analysis using various databases and web tools to identify key DEGs and investigate the prognostic value of these hub genes in STAD. Moreover, we evaluated the gene alteration status of hub genes and investigated the association between hub genes and immune cell infiltration in STAD.

## 2. Materials and Methods

### 2.1. Identification of Differentially Expressed mRNAs

GEPIA (http://gepia.cancer-pku.cn/) is a web-based tool delivering rapid and customizable functionalities based on data from The Cancer Genome Atlas and Genotype-Tissue Expression. This tool provides key interactive functions corresponding to differential expression analysis, profile plotting, correlation analysis, patient survival analysis, similar gene detection, and dimensionality reduction analysis [15]. The Cancer RNA-Seq Nexus (CRN; http://syslab4.nchu.edu.tw/index.jsp) is an open resource for obtaining coding-transcript profiles and identifying differentially expressed mRNAs to support researchers in generating new ideas in cancer research [16]. In this study, we initially used the GEPIA database to identify DEGs and survival-related genes. mRNAs with *q* values < 0.01 and ∣log2FC | ≥2 were considered DEGs. We further screened the DEGs using the CRN online software, and *P* value < 0.01 was set as the cut-off standard.

### 2.2. Confirmation of the Expression Level of Key Genes by UALCAN

The common genes were selected among GEPIA identified DEGs, CRN identified DEGs, and survival-related DEGs using the Venn diagram, and these common genes were named as hub genes [17]. Moreover, we used the UALCAN (http://ualcan.path.uab.edu/index.html database) to validate the expression of key genes in STAD based on different clinical characteristics, including tumor stage, tumor grade, and nodal metastasis status [18].

### 2.3. Survival Analysis of the Hub Genes

The Oncolnc (http://www.oncolnc.org/) and HPA databases (http://www.proteinatlas.org/) are user-friendly web resources for analyzing the association between genes and prognosis. We investigated the influence of the hub genes on prognosis through the Oncolnc and Human Protein Atlas (HPA) databases [19, 20].

### 2.4. Gene Alteration of Three Hub Genes from cBioPortal Database

The cBio Cancer Genomics Portal (http://cbioportal.org) is an online resource for the exploration of multidimensional cancer genomics data sets [21]. In this study, the cBioPortal database was used to analyze the types and frequency alteration of three hub genes (e.g., mutation, amplification, deep deletion, and multiple alterations) in three hub genes.

### 2.5. Immune Infiltration in STAD with Different Somatic Copy Number Alterations (SCNAs)

TIMER is a publicly available resource for the systematic analysis of immune infiltrates across different types of cancer (https://cistrome.shinyapps.io/timer/). It contains seven modules, including gene, survival, mutation, SCNA, differential gene expression, correlation, and estimation. The SCNA module compares the levels of infiltration among tumors with different SCNAs in a given gene. SCNAs are divided into five types, namely, deep deletion (−2), arm-level deletion (−1), diploid/normal (0), arm-level gain (1), and high amplification (2). Box plots show the distribution of each immune subset at each copy number status in selected types of cancer. The level of infiltration for each SCNA category is compared with the normal using the two-sided Wilcoxon rank-sum test [9, 22]. In this study, we evaluated the level of immune infiltration for each SCNA category in three hub genes using the SCNA module.

### 2.6. Association between Immune Cell Infiltration and Hub Genes

The gene module of the TIMER allows the user to visualize the correlation between gene expression and infiltration levels of immune cells in different types of cancer [22]. In this study, we used the gene module to evaluate the correlation between immune infiltrates and three hub genes. Moreover, GEPIA database was applied to confirm the correlation between three hub genes expression and immune cell infiltration.

## 3. Results

### 3.1. Identification of DEGs

**Figure 1:**
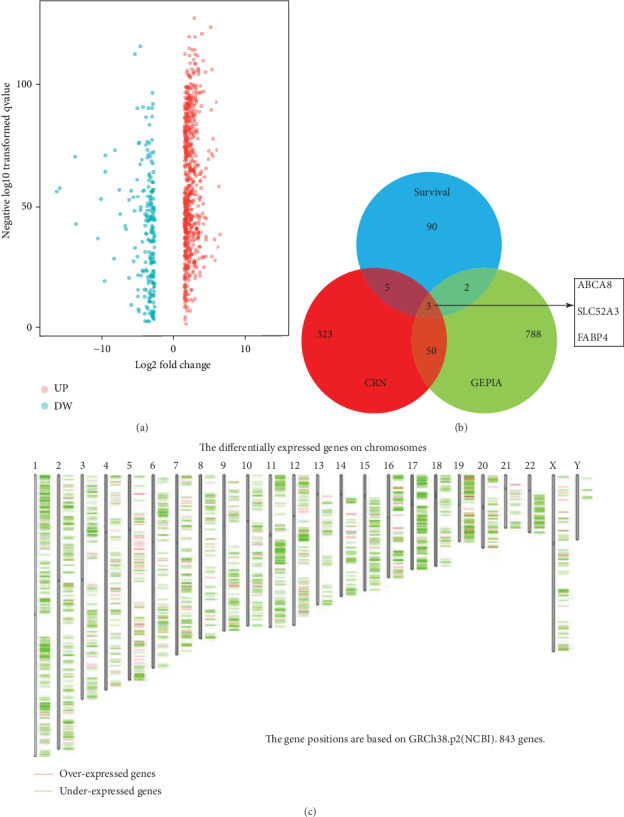
The differentially expressed genes in STAD. (a) Volcano plot of differently expressed genes between STAD and normal stomach tissues. (b) Venn diagrams were used to show the common genes. (c) Chromosomes distribution of DEGs in GEPIA database.

A total of 843 DEGs were identified from the GEPIA database, including 638 upregulated genes and 205 downregulated genes (Figure 1(a)). 381 DEGs were identified in the CRN database. 100 survival-related DEGs were obtained from the GEPIA database. Three common hub genes were obtained, including 2 downregulated DEGs (ABCA8 and FABP4) and one upregulated DEG (SLC52A3) (Figure 1(b)). We further analyzed the chromosomal distribution of DEGs in the GEPIA database. Our results showed that the 843 DEGs were distributed in 22 pairs of autosomes and one pair of sex chromosomes (Figure 1(c)).

### 3.2. Confirmation Key Genes Expression Based on Different Clinical Characteristics

**Figure 2:**
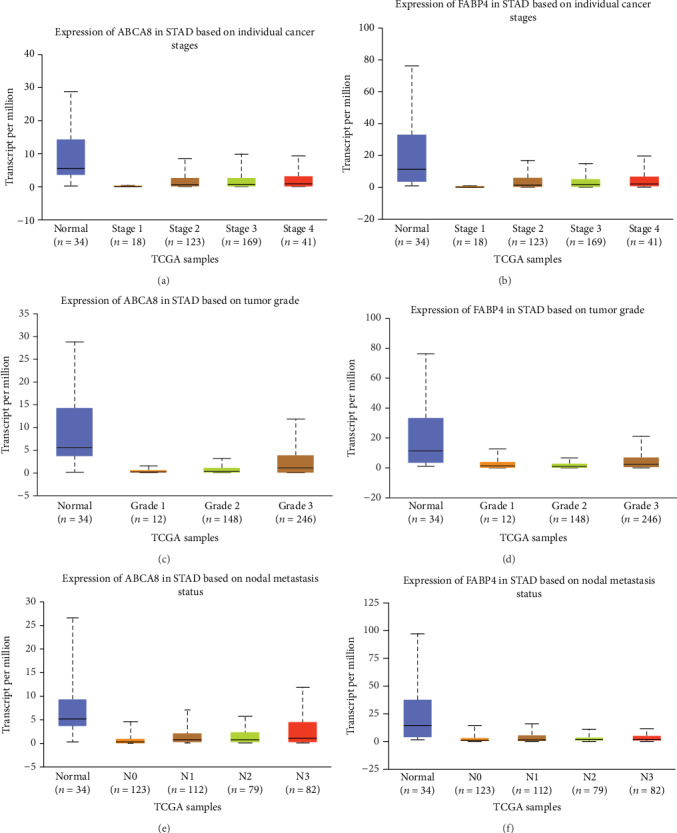
Confirmation of the expression level of key genes by UALCAN. We further examined the expression of three hub genes under different conditions using the UALCAN database.

We further examined the expression of three hub genes under different clinical characteristics using the UALCAN database. Our results showed that ABCA8 and FABP4 expression were downregulated in STAD compared with normal tissues. Patients with advanced STAD exhibited higher expression of ABCA8 and FABP4 (Figures 2(a) and 2(b)). ABCA8 and FABP4 expression levels were significantly increased in higher grade patients (Figures 2(c) and 2(d)). The highest expression level of ABCA8 and FABP4 was detected in N3 (Figures 2(e) and 2(f)).

### 3.3. Evaluation of the Relationship between Hub Genes Expression and Prognosis

**Figure 3:**
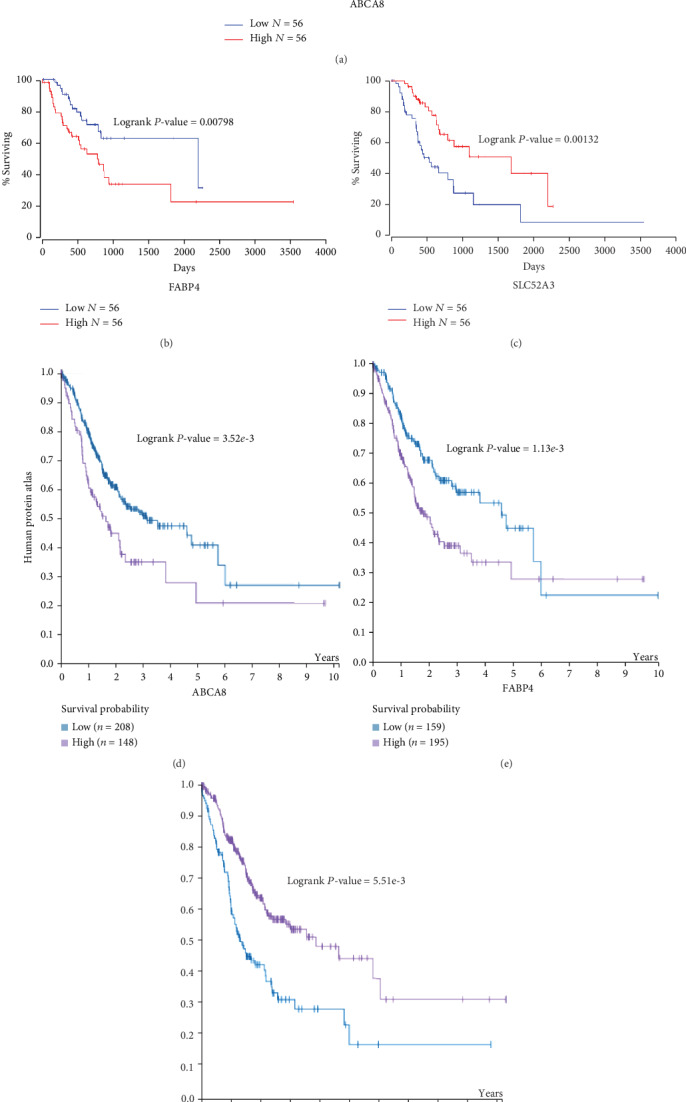
Correlation between hub genes expression and patient survival in STAD. The Oncolnc and HPA databases were used to analyze the association between the expression of the three key genes and survival.

The Oncolnc and HPA databases were applied to elucidate the prognostic value of three hub genes in STAD. The results indicated that high ABCA8 and FABP4 expression were associated with poor prognosis in STAD (*P* < 0.05; Figures 3(a), 3(b), 3(d), and 3(e)). As shown in Figures 3(c) and 3(f), the survival probability of patients in the SLC52A3 high expression group was higher than those in the low expression group (*P* < 0.05). These results suggested that three hub genes can be considered as prognostic biomarkers for patients with STAD.

### 3.4. Genetic Alteration of Three Hub Genes

**Figure 4:**
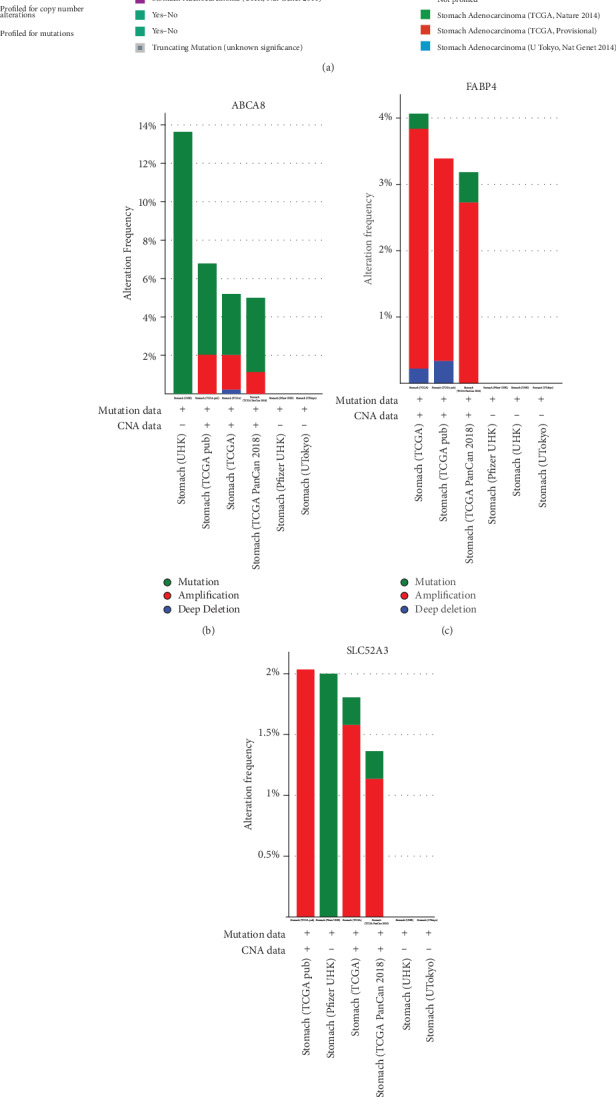
Genetic alteration of three hub genes. (a) The cBioPortal database was applied to analyze the genetic alteration of three hub genes. The genetic alteration, including mutation, amplification, deep deletion, and multiple alterations. (b–d) The genetic alteration types and frequency of three hub genes in STAD were examined by the cBioPortal database.

We used the cBioPortal database to investigate the genetic alteration of ABCA8, FABP4, and SLC52A3 in 1365 STAD samples. The results showed genetic alteration rates of ABCA8, FABP4, and SLC52A3 in STAD were 5%, 3%, and 1.7%, respectively (Figure 4(a)). Genetic alteration types and frequency of these three hub genes showed differences in various STAD (Figures 4(b)–4(d)). Our results suggested that genetic alterations of these three hub genes may play an important role in STAD.

### 3.5. The Association of SCNAs of Hub Genes with Immune Infiltration Was Different

**Figure 5:**
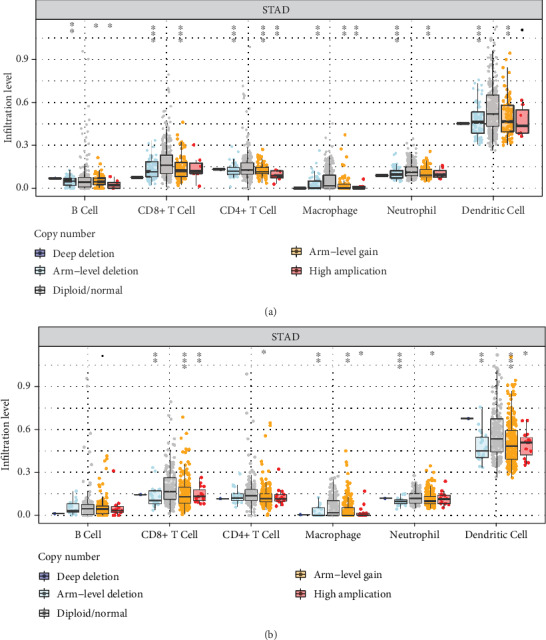
Correlation between SCNA of hub genes and immune infiltration in STAD. The somatic copy number alterations (SCNA) module of TIMER was used to evaluate the correlation between SCNA of hub genes and immune infiltration in STAD. SCNA of hub genes are defined by GISTIC 2.0, including deep deletion, arm level deletion, normal, arm-level gain, and high amplification. (a) and (b) showed that the immune cell enrichment was decreased in STAD with different SCNAs of ABCA8 and FABP4. Box plots represent the distributions of each immune subset at each copy number status in STAD. The infiltration level for each SCNA category was compared with the normal using a two-sided Wilcoxon rank sum test.

The SCNA module of the TIMER was performed to examine the association between the SCNAs of three hub genes and immune infiltration. The results showed that the immune cell enrichment was decreased in STAD with different SCNAs of ABCA8 and FABP4. The association between SCNAs of SLC52A3 and immune cell infiltration was not identified (Figures 5(a) and 5(b)). Our results suggested that the genetic alteration of ABCA8 and FABP4 is closely associated with the enrichment of immune cell infiltration in STAD.

### 3.6. Correlation between Hub Genes and Immune Cell Infiltration in STAD

**Figure 6:**
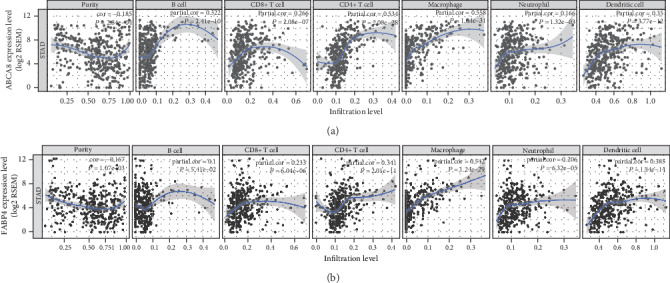
Correlation between three hub genes and immune cell infiltration in STAD. (a) ABCA8 is negatively associated with tumor purity and is positively correlated with B cells, CD4+ T cells, CD8+ T cells, Neutrophils, Macrophages, and Dendritic cells. (b) FABP4 is negatively associated with tumor purity and is positively correlated with above mentioned six immune cells. Both ABCA8 and FABP4 are positively correlated with macrophages. SLC52A3 is not be identified by TIMER.

The gene module of the TIMER was applied to evaluate the association between the expression of these three hub genes and immune infiltration in STAD. The results indicated that ABCA8 and FABP4 were negatively correlated with tumor purity and were positively associated with six types of immune cells. This is particularly true for infiltrated macrophages. The association between the SLC52A3 expression and immune cell infiltration was not detected using TIMER (Figures 6(a) and 6(b)). We further confirm the correlation between three hub genes expressions and M1 and M2 macrophages infiltration using the GEPIA web tool. Our data showed that ABCA8 and FABP4 expression were positively correlated with M2 macrophages in STAD (Tables 1 and 2). Our results suggested that ABCA8 and FABP4 may affect the infiltration of immune cells, especially on M2 macrophages.

## 4. Discussion

STAD is the second leading cause of cancer-related death due to its poor prognosis [2, 3]. The identification of a new valuable prognostic marker for STAD becomes more urgent [23]. In recent years, a growing body of evidence indicates that gene expression and immune cell infiltration play a key role in the prognosis of tumors [1, 5]. However, the association between gene expression, the infiltration of immune cells, and prognosis has not been completely understood.

ABCA8, a member of the superfamily of ATP-binding cassette transporters, plays a critical role in cancer biology and drug resistance [24]. ABCA8 is mostly downregulated in different types of cancers, including hepatocellular carcinoma, prostate cancer, ovarian cancer, and tongue squamous cell carcinoma [25–28]. High expression of ABCA8 in various cancers has been reported to be correlated with poor prognosis [27, 29]. However, the overexpression of ABCA8 has been found to be correlated with the favorable prognosis of patients with hepatocellular carcinoma [25]. FABP4 (Fatty Acid Binding Protein 4) is a Protein Coding gene. High expression of FABP4 contributes to the poor prognosis in Ovarian Carcinoma, nonsmall cell lung cancer, Pancreatic Ductal Adenocarcinomas, and hepatocellular carcinoma [30–33]. There was a significant correlation between FABP4 expression and tumor grade [34]. This study demonstrated that ABCA8 and FABP4 were downregulated in STAD, and high expression of ABCA8 and FABP4 was associated with poor prognosis. We further confirmed that ABCA8 and FABP4 expression were significantly decreased in STAD tissues, regardless of clinical characteristics, such as cancer stage, grade, and nodal metastasis status of STAD. In addition, we used the cBioPortal database to investigate the genetic alteration of ABCA8 and FABP4 in 1365 STAD samples. Our results suggested that genetic alterations of these hub genes may play an important role in STAD. Taken together, it suggests that the expression of ABCA8 and FABP4 may predict the prognosis of STAD.

Human riboflavin transporter-3 (encoded by SLC52A3) encodes a riboflavin transporter protein that plays an important role in the intestinal absorption of riboflavin and affects the distribution of riboflavin in tissue [35]. In a previous study, SLC52A3 was identified as a prognostic biomarker in esophageal cancer [36]. There is not any report about the association between SLC52A3 expression and prognosis in STAD patients. Our results indicated that SLC52A3 was upregulated in STAD, and high expression of SLC52A3 was associated with favorable prognosis.

Previous findings have shown that immune cell infiltration is related to the prognosis of STAD [1, 13]. A number of studies have shown that high infiltration of M2 macrophages indicates poor prognosis [1, 37–39]. In Cervical Cancer, FABP4 was considered to be correlated to immune cell infiltration [40]. To explore factors related to patient prognosis that is regulated by hub genes expression, association between hub genes expression and immune cell infiltration was analyzed. Our data demonstrated that ABCA8 and FABP4 expression were positively associated with six types of immune cells, especially in M2 macrophages. Our data showed immune cell enrichment was decreased in STAD with different SCNAs of ABCA8 and FABP4. Taken together, our findings suggested that increased ABCA8 and FABP4 expression predict poor prognosis in STAD and are associated with immune cell infiltration. Especially, the infiltration of M2 macrophages cells may affect the prognostic value of ABCA8 and FABP4 expression.

## 5. Conclusion

In summary, the findings of the present study showed that three hub genes were associated with the prognosis of STAD. Genetic alterations of these three hub genes may play a significant role in STAD. ABCA8 and FABP4 were correlated with immune infiltration and displayed a positive correlation with M2 macrophages. Our results suggest that ABCA8 and FABP4 can be used as prognostic biomarkers, and the infiltration of M2 macrophages cells that may affect the prognostic value of ABCA8 and FABP4 expression. Although these data provide insight into the roles of three hub genes in STAD, the in vivo or in vitro experiment also needs to be performed to verify our above results.

## Figures and Tables

**Table 1:** Correlation analysis between ABCA8 and gene markers of macrophages using GEPIA.

Description	Gene marker	STAD	
		Tumor	
		*R*	*P*
M1 macrophages	INOS(NOS2)	-0.078	0.11
	IRF5	0.098	0.048
	COX2(PTGS2)	0.066	0.19
M2 macrophages	CD163	0.23	2.1*E*-6
	VSIG4	0.24	1.6e-6
	MS4A4A	0.37	6.2*e*-15

**Table 2:** Correlation analysis between FABP4 and gene markers of macrophages using GEPIA.

Description	Gene marker	STAD	
		Tumor	
		*R*	*P*
M1 macrophages	INOS(NOS2)	-0.047	0.34
	IRF5	-0.024	0.63
	COX2(PTGS2)	0.031	0.53
M2 macrophages	CD163	0.13	0.091
	VSIG4	0.17	0.00047
	MS4A4A	0.13	0.011

## Data Availability

The Gene Expression Profiling Interactive Analysis (GEPIA) and Cancer RNA-Seq Nexus (CRN) database used to support the findings of this study are available at (http://gepia.cancer-pku.cn/) and (http://syslab4.nchu.edu.tw/index.jsp respectivly). The data of these two online analysis databases are from TCGA database (https://cancergenome.nih.gov/).

## Abbreviations

GC: Gastric cancer
STAD: Stomach adenocarcinoma
DEGs: Differentially expressed genes
GEPIA: Gene expression profiling interactive analysis
CRN: The Cancer RNA-Seq Nexus
cBioPortal: The cBio Cancer genomics portal
SCNA: Copy number alterations.
